# How to Evaluate Acne in Reproductive-Age Women: An Epidemiological Study in Chinese Communities

**DOI:** 10.1155/2019/6126808

**Published:** 2019-02-03

**Authors:** Yu-Yang Wang, Shang-Wei Li, Shan Luo, Lang Qin, Xun Zeng, Lei Li, Xiao-Hong Li

**Affiliations:** ^1^Nursing Department of West China Second University Hospital, Sichuan University, Chengdu, China; ^2^Key Laboratory of Birth Defects and Related Diseases of Women and Children, Sichuan University, Ministry of Education, Chengdu, China; ^3^Department of Obstetrics and Gynecology, West China Second University Hospital, Sichuan University, Chengdu, China

## Abstract

**Background:**

Acne is not only a skin condition but also a cardinal component of many systemic diseases or syndromes. This study was aimed to investigate the prevalence of acne in reproductive-age women in Sichuan province, China, and to evaluate acne as a skin problem alone or a symptom of gynecological/endocrinological disease.

**Methods:**

From October 2008 to September 2009, 1043 reproductive-age women from 19 to 45 years of age from seven communities of three districts in Sichuan province completed a standardized questionnaire and a physical examination. Acne was classified using the Pillsbury scale, and hirsutism was assessed using a modified Ferriman–Gallwey method. Diagnosis of polycystic ovary syndrome (PCOS) was based on the 2003 Rotterdam criteria. Some endocrine and metabolic markers were detected for the women diagnosed with PCOS related to acne and the control group.

**Results:**

The prevalence of acne was 32.5%, and the highest prevalence (9.6%) was seen in the 19–24-year-old age group. Prevalence among women eating dessert frequently, exercising seldom, or among sedentary workers was significantly higher in the acne group (14.1%, 55.6%, and 51.3%, respectively) than in the nonacne group (10.8%, 45.7%, and 35.5%; all P<0.05). The prevalence of oligomenorrhea and hirsutism in the acne group (17.6%, 24.7%) was significantly higher than in the nonacne group (8.6%, 15.1%; both P<0.05). Among the participants with acne, 64.3% had acne alone, 18.3% were diagnosed with hyperandrogenism, and 17.4% were diagnosed with PCOS. The level of serum androstendione in the group of PCOS (10.98±3.12 nmol/L) was significantly higher than that in the control group (8.85±3.09nmol/L) (P<0.05).

**Conclusion:**

When reproductive-age women with acne are encountered in gynecology–endocrinology or dermatology clinics, physicians should consider evaluating them from PCOS, hyperandrogenism, or acne alone.

## 1. Background 

Acne vulgaris is a chronic inflammatory disease of the pilosebaceous unit, characterized by seborrhea, formation of comedones, erythematous papules, and pustules [1]. It affects nearly 80 percent of people at some time between the ages of 11 and 30 years. It can persist for several years and result in disfigurement and permanent scarring, and it can have serious adverse effects on psychosocial development, resulting in emotional problems, withdrawal from society, and depression [2]. The precise pathogenesis of acne vulgaris has remained enigmatic, but the classic concept is that it results from the combination of increased sebaceous gland activity with seborrhea, abnormal follicular differentiation with increased keratinization, microbial hypercolonization of the follicular canal, and increased inflammation, primarily through activation of the adaptive immune system [3]. A significant role of androgens is also recognized [4]. Acne, hirsutism, and androgenetic alopecia are classically considered signs of cutaneous hyperandrogenism. These common skin findings have posed the diagnostic challenge of excluding underlying disorders such as polycystic ovary syndrome (PCOS) [5].

PCOS is the most common and concerning endocrine disorder in reproductive-age women and was first described by Stein and Leventhal as a syndrome of oligo/amenorrhea and polycystic ovaries, which was variably accompanied by hirsutism, acne, and obesity [6]. It affects not only women's menstrual cycles and reproductive function but also their emotions and can be associated with moderate or severe depression and anxiety symptoms [7]. According to the National Institute of Health meeting in 1990, the Rotterdam consensus workshop group meeting in 2003, and the Androgen Excess-PCOS Society Task Force in 2006, the clinical signs of hyperandrogenism or biochemical hyperandrogenism are very important diagnostic criteria for PCOS [8]. Cutaneous manifestations might be the first signs of PCOS, so women presenting with acne and/or hirsutism should be evaluated in terms of PCOS [9, 10].

Hirsutism is a common manifestation of hyperandrogenism [11] and is the most reliable cutaneous marker of PCOS [5]. Acne was a prevalent but unreliable marker of biochemical hyperandrogenism in one PCOS study population [5]. However, not all acne is associated with androgen excess. Demir et al. found that approximately 50% of normal women with acne did not have clinical or biochemical evidence of hyperandrogenism. Ozdemir et al. inferred that acne is not associated with hormonal variables [12]. Schmidt suggested that androgen action at the target organ level in both dermatoses might be independent of peripheral serum levels of hormones [13].

Thus, acne is not only a skin condition but can be a cardinal component of many systemic diseases or syndromes [14]. However, no previous paper has described how to distinguish acne as a skin condition alone from acne as a symptom of other disorders. The aims of this study were to understand the prevalence of acne in reproductive-age women in the province of Sichuan in China and to conduct an epidemiological evaluation of acne as either an isolated skin problem, or a symptom of hyperandrogenism, or PCOS.

## 2. Materials & Methods 

### 2.1. Study Population

From October 2008 to September 2009, we carried out an epidemiological investigation of reproductive-age women, aged 19 to 45 years, in Sichuan province, China. This investigation was approved by the Medical Ethics Committee of West China Second Hospital, Sichuan University (Permit Number: 2008081). A total of 1043 women from seven communities, including rural and urban communities in three districts, were surveyed in the study. All participants were of Han ethnicity. All gave written informed consent and underwent a free medical evaluation. Two interviewers from our university hospitals were trained to use a standardized questionnaire and conduct a physical examination.

### 2.2. Study Protocol

Participants were asked to answer questions in a previously validated questionnaire [15] with the instruction of trained investigators. The questions included detailed menstrual history, reproductive history, work and life habits, skin problems, and endocrine and metabolic diseases. A transvaginal ultrasound scan of follicle numbers and ovarian volume was included in the participants' clinical examination. Fasting blood from the women diagnosed with PCOS related to acne and the control women who were matched with 1:2 was detected androstendione (A), testosterone (T), sex hormone binding globulin (SHGB), insulin (INS), thyroid-stimulating hormone (TSH), cholestenone (CHO), and triglyceride (TG).

Acne was defined by the presence of comedones, papules, pustules, nodules, cysts, and scars on the face, neck, and upper trunk. The presentation of scars alone was not included. The severity of acne was classified using a three-point grading scale. Mild acne was defined as grade I of the Pillsbury scale [16]. Moderate acne included grades II and III. Severe acne was grade IV.

The amount of excess terminal hair growth was assessed using a modified Ferriman–Gallwey (mF-G) method, scoring the presence of terminal hairs over nine body areas (upper lip, chin, chest, upper and lower abdomen, thighs, upper and lower back, and upper arms) from 0 to 4 [17].

The diagnosis of PCOS was based on the 2003 Rotterdam consensus workshop, including at least two of three criteria: (1) oligomenorrhea and/or anovulation (eight or fewer menstrual cycles in 1 year or menstrual cycles >35 days in length); (2) clinical and/or biochemical signs of hyperandrogenism; (3) polycystic ovaries (the presence of 12 or more follicles in each ovary measuring 2–9 mm in diameter and/or increased ovarian volume of >10 mL); and exclusion of other etiologies (for example, congenital adrenal hyperplasia, androgen-secreting tumors, or Cushing's syndrome) [18].

### 2.3. Statistical Analysis

Chi-squared tests were used to compare the categorical variables, such as prevalence of oligomenorrhea, and hirsutism. Student's t-tests were used to compare the numerical variables, such as body mass index (BMI), waist–hip ratio (WHR), and androstendione. All statistical analyses were performed using SPSS version 17.0 (IBM Corp., Armonk, NY, USA) and significance was defined as P < 0.05.

## 3. Results

### 3.1. Study Population

A total of 1043 female participants from an original sample of 1104 completed the questionnaire and received a physical examination. The age of the study population ranged from 19 to 45 years of age (mean 37 years).

### 3.2. Prevalence of Acne

Among the 1043 participants, 339 (32.5%) were found to have acne. The severity of acne was mild in 246 participants (23.6%) and moderate in 93 (8.9%). No severe acne was found in our investigation. When we divided the participants into 5-year age groups, the highest prevalence was seen in the 19–24-year-old group (9.6%). The prevalence of acne was 5.1% in the 25–29-year-old group, 6.9% in the 30–34-year-old group, 6.1% in the 35–39-year-old group, and 4.8% in the 40–45-year-old group.

### 3.3. Risk Factors for Acne

To study the relationship between acne and menstrual cycling, we divided the participants into acne and nonacne groups and their menstrual cycling into <21 days, 21–35 days, and >35 days. We defined a menstrual cycle of >35 days as oligomenorrhea. There were 121 participants (11.6%) who had oligomenorrhea, and the prevalence of oligomenorrhea in the acne group (17.6%) was higher than in the nonacne group (8.6%; P<0.05). There was no difference in the prevalence of menstrual cycles <21 days or 21–35 days in the acne versus nonacne groups. (Table 1)

There were 349 participants (33.5%) who had an mF-G score of 0, 503 (48.2%) who had an mF-G score of 1, and 191 (18.3%) who had an mF-G score ≥2. The prevalence of an mF-G score ≥2 in the acne group (24.7%) was higher than in the nonacne group (15.1%; P<0.05; Table 2)

We divided the frequencies of nonstaple foodstuff consumption and exercise participation into “seldom,” “sometimes,” and “frequently.” We defined frequently as ≥3 times per week, seldom as less than once per month, and sometimes as intermediate between the two. There were 49 participants (14.1%) who ate dessert frequently in the acne group, which was higher than in the nonacne group (10.8%; P<0.05). There was no difference in fried food consumption between the acne and the nonacne group. There were 193 participants (55.6%) who seldom exercised in the acne group, which was higher than in the nonacne group (45.7%; P<0.05; Table 3). In the acne group, there were 178 sedentary workers (51.3%), which were higher than in the nonacne group (35.5%; P<0.05).

There were no differences in weight gain over the last three years, BMI, waistline, and WHR in the acne versus the nonacne groups (Table 4).

### 3.4. PCOS Related to Acne

Among the 339 participants who had acne, there were 59 women (17.4%) with oligomenorrhea (with or without hirsutism) who were diagnosed with PCOS, 62 (18.3%) with hirsutism who were diagnosed with hyperandrogenism, and 218 (64.3%) who had acne alone. The level of serum androstendione in the group of PCOS (10.98±3.12 nmol/L) was significantly higher than that in the control group (8.85±3.09nmol/L) (P<0.05). There were no differences between groups in relation to serum T, SHBG, INS, TSH, CHO, and TG (Table 5).

In our investigation, we only found polycystic ovaries by transvaginal ultrasound in 6 women. Because of the small samples, we did not perform statistics analysis.

## 4. Discussion

Acne vulgaris is a common skin condition. In our investigation, we focused on women of childbearing age (from 19 to 45 years of age) in seven communities of three districts in Sichuan province. We found that the total prevalence of acne was 32.5%, and the highest prevalence (9.6%) was seen in the 19–24-year-old group. With increasing age, the prevalence of acne decreased and prevalence in the 40–45-year-old group was 4.8%. The specific prevalence of acne was not the same as previous large studies in the UK, USA, and China, but the linear trend inversely proportional to age was similar [1, 19, 20]. In the previous large-scale Chinese study, six cities were investigated including one district of Sichuan province, but this district was not included in our investigation. The prevalence of acne is thought to vary among ethnic groups, geographical zones, and countries [21].

We found that participants with acne tended to eat dessert frequently. The relationship between acne and diet is controversial. Shen et al. reported that no association was found between greasy/spicy diet and acne [1]. However, an increasing number of studies provide compelling evidence that high-glycemic-load diets may exacerbate acne [22, 23], and a reduction in glycemic load for 10 weeks has been reported to improve acne [24]. We also observed that women with acne seldom exercised compared to women without acne. Although the relevant literature is limited, Short et al. reported that exercise-induced sweat had no significant effect on truncal acne [25]. The relationship between acne and exercise could be explored in prospective clinical studies in the future. In addition, we found that there were no significant associations between acne and BMI or WHR, which is similar to previous studies [26, 27].

Acne, hirsutism, and oligomenorrhea are the important diagnostic features of PCOS. Pembe and Abeid reported that oligomenorrhea and acne were significantly higher in a group of women with PCOS than a group of women with normal ovaries [28]. In our study, among the participants with acne, 17.4% women were diagnosed with PCOS because they also had oligomenorrhea (with or without hirsutism). The levels of serum androstendione in the group of PCOS were significantly higher than that in the control group. Hasinski et al. also reported that women with acne and oligomenorrhea have higher levels of biologically active testosterone than those with normal menses [29]. The hyperandrogenism of PCOS appears to be aptly multifactorial, and the effectiveness on hyperandrogenic symptoms by antiandrogens may or may not be reflected by the suppression of serum androgens [30].

## 5. Conclusions

Our evidence-based data should help clinicians to treat acne patients. We found that the prevalence of acne was 32.5% in reproductive-age women from 19 to 45 years of age. Among them, 17.4% were diagnosed with PCOS, 18.3% were diagnosed with hyperandrogenism, and 64.3% had acne alone. Thus, when reproductive-age women with acne are encountered in gynecology–endocrinology clinics or dermatology clinics, physicians should consider evaluating them from PCOS, hyperandrogenism, or acne alone and give them an appropriate therapy.

## Figures and Tables

**Table 1:** The women's menstrual cycle in the acne and nonacne group.

Group	≤21days	21-35 days	≥35days^*∗*^
	%(n)	%(n)	% (n)
Acne group	6.3% (22)	76.1% (264)	17.6% (61)
Non-acne group	5.7% (40)	85.7% (596)	8.6% (60)

Data are shown as rates and were analyzed by *χ*^2^ test.  ^*∗*^P<0.05.

**Table 2:** The women's mF-G score in the acne and nonacne group.

Group	0	1	≥2^*∗*^
	%(n)	%(n)	% (n)
Acne group	29.9% (104)	45.4% (158)	24.7% (86)
Nonacne group	35.3% (245)	49.6% (345)	15.1% (105)

Data are shown as rates and were analyzed by *χ*^2^ test.  ^*∗*^P<0.05.

**Table 3:** The women's nonstaple foodstuff and exercise habit in the acne and nonacne group.

	Dessert %(n)			Fried food %(n)			Exercise %(n)		

Group	Seldom	Sometimes	Frequently	Seldom	Sometimes	Frequently	Seldom	Sometimes	Frequently
Acne group	22.2%(77)	63.7%(221)	14.1%(49)^*∗*^	15.6%(54)	68.9%(239)	15.5%(54)	55.6%(193)^*∗*^	44.4%(154)	0
Non-acne group	17.9%(125)	71.3%(496)	10.8% (75)	14.5%(101)	72.9%(508)	12.5%(87)	45.7%(318)	54.2%(377)	0.1%(1)

Data are shown as rates and were analyzed by *χ*^2^ test. ^*∗*^P<0.05.

**Table 4:** The women's weight in the acne and nonacne group.

	Gain weight in late 3 years %(n)^a^			WHR^b^	BMI(kg/m2)^c^
Group	≤5kg	5-10kg	≥10kg		
Acne group	85.3% (296)	11.0% (38)	3.7% (13)	0.83±0.10	22.4±5.0
Non-acne group	87.2% (607)	11.5% (80)	1.3% (9)	0.83±0.09	22.8±4.8

^a^Data are shown as rates and were analyzed by *χ*^2^ test. ^b.c^Data are shown as the mean ± SD and were analyzed by t test.

**Table 5:** The endocrine and metabolic assessment of PCOS group related to acne and non-PCOS group.

Group	N	A^*∗*^	T	SHBG	INS (mIU/L)	TSH (mIU/L)	CHO	TG (mmol/L)
		(nmol/L)	(nmol/L)	(nmol/L)			(mmol/L)	
PCOS group	59	10.98±	1.74±	65.7±	4.61±	2.48±	4.69±	1.27±
		3.12	0.78	30.9	1.91	1.33	2.01	0.62
Control group	118	8.85±	1.41±	67.35±	3.95±	2.93±	4.36±	1.06±
		3.09	0.67	29.8	2.02	1.42	2.37	0.49

Data are shown as the mean ± SD and were analyzed by t test.  ^*∗*^P<0.05.

## Data Availability

The data used to support the findings of this study are included within the article.
